# Recharging Difficulty With Pulse Generator After Deep Brain Stimulation: A Case Series of Five Patients

**DOI:** 10.3389/fnins.2021.705483

**Published:** 2021-09-27

**Authors:** Hongyang Li, Daoqing Su, Yijie Lai, Xinmeng Xu, Chencheng Zhang, Bomin Sun, Dianyou Li, Yixin Pan

**Affiliations:** ^1^Department of Neurosurgery, Ruijin Hospital, Shanghai Jiao Tong University School of Medicine, Shanghai, China; ^2^Center for Functional Neurosurgery, Ruijin Hospital, Shanghai Jiao Tong University School of Medicine, Shanghai, China; ^3^Department of Neurosurgery, Liaocheng People’s Hospital, Liaocheng Clinical School of Shandong First Medical University, Liaocheng, China

**Keywords:** hardware complication, deep brain stimulation, implanted pulse generator, rechargeable battery, Parkinson’s disease

## Abstract

**Background:** Deep brain stimulation (DBS) is a well-established treatment for a variety of movement disorders. Rechargeable cell technology was introduced to pulse generator more than 10 years ago and brought great benefits to patients. However, with the widespread use of rechargeable implanted pulse generators (r-IPGs), a new hardware complication, when charging the r-IPG has been difficult, was encountered.

**Objective:** The aims of this study were to report five cases confronted with r-IPG charging difficulty postoperatively and to explore the predisposing factors and treatment strategies for this rare complication.

**Methods:** We retrospectively reviewed our DBS patient database for those who were implanted with r-IPGs. From 2012, we identified a total of 1,226 patients, with five of them experiencing charging difficulties after surgery. Detailed patient profiles and clinical procedures were scrutinized and reviewed.

**Results:** All the charging problems were resolved by reoperation. Cases 1 and 2 required their r-IPGs to be anchored to the muscle and fascia. Cases 3 and 4 had their r-IPGs inserted in the wrong orientation at the initial surgery, which was resolved by turning around the r-IPGs at the revision surgery. Case 5, in which we propose that the thick subcutaneous fat layer blocked the connection between the r-IPG and the recharger, required a second operation to reposition the r-IPG in a shallow layer underneath the skin. For all cases, the charging problems were resolved without reoccurrences to date.

**Conclusion:** Our case series indicates a novel hardware complication of DBS surgery, which had been rarely reported before. In this preliminary study, we describe several underlying causes of this complication and treatment methods.

## Introduction

Deep brain stimulation (DBS) has been shown to be a safe and effective treatment for a variety of movement disorders such as Parkinson’s disease (Zhang et al., 2020b) and dystonia (Krauss et al., 2021) and neurobehavioral disorders like Tourette syndrome (Xu et al., 2020). The introduction of rechargeable implanted pulse generators (r-IPGs) in the 2000s has brought great benefits to DBS patients (Zhang et al., 2020a), including prolonging the life of the pulse generators and reducing the time of IPG replacement surgeries (Qiu et al., 2021), thereby alleviating the suffering of DBS patients and lowering the associated risks of potential infections (Thrane et al., 2014). Furthermore, a reduced long-term cost is achievable when investing in a r-IPG (Rizzi et al., 2015).

However, in clinical practice, a novel hardware-related complication wherein charging the r-IPGs had been difficult has been indicated. In this article, we describe five patients who presented with r-IPG charging difficulties postoperatively and anticipate discovering the underlying causes of and treatment methods for this complication.

## Materials and Methods

We retrospectively reviewed our DBS patient database at the Department of Functional Neurosurgery, Ruijin Hospital, between January 2012 and January 2021, which included 1,226 patients who were implanted with r-IPGs. Among the 1,226 patients, charging problems emerged in five of them and further revision surgeries were noted. Detailed medical records were collected and scrutinized. This study was approved by the Ethics Committee of the Ruijin Hospital, Shanghai Jiao Tong University School of Medicine. Written informed consent for the study was exempted by the Ethical Committee due to its retrospective nature and anonymous data presentation.

## Results

### Clinical Characteristics

The clinical characteristics of the five cases included in our case series are summarized in Table 1. Three patients were diagnosed with Parkinson’s disease (PD) and the other two with Tourette syndrome. The rechargeable implanted pulse generator (IPG) models in three patients were Activa RC (model 37612), manufactured by Medtronic (Minneapolis, MN, United States), and G102R, manufactured by PINS (Beijing, China), for the other two. The average time for the onset of complications was 13.8 (2–35) months following implantation.

**TABLE 1:** Clinical characteristics of the recharging difficulty presented by patients in this article, along with those presented in the literature for comparison.

Author and publication year	Indication	Sex/age (years)	DBS equipments	Etiology	Occurrence after initial surgery	Diagnosis	Treatment
Chelvarajah et al. (2012)	Hemidystonia	F, 19	Medtronic	Migration of the adaptor to lie superficially on the r-IPG	8 months	Palpation, X-ray	Manual manipulation, restoring the adaptor beneath the IPG, reducing the pocket size
	Generalized dystonia	F, 16	Medtronic		4 months	Palpation, X-ray	Manual manipulation
Case 1	Tourette syndrome	F, 32	Medtronic	Weight loss, intentional manipulation of the IPG	18 months	X-ray findings, patient history	Anchoring the IPG to the pectoralis fascia, reducing the pocket size
Case 2	Parkinson’s disease	F, 71	Medtronic	Improper fixation of the IPG	12 months	X-ray findings, patient history	Anchoring the IPG to the pectoralis fascia, reducing the pocket size
Case 3	Parkinson’s disease	F, 66	PINS	Wrong orientation of IPG insertion	2 months	X-ray findings, patient history	Reinsertion of the IPG at the correct orientation
Case 4	Parkinson’s disease	F, 62	PINS	Wrong orientation of IPG insertion	3 days	X-ray findings, patient history	Reinsertion of the IPG at the correct orientation
Case 5	Tourette syndrome	M, 23	Medtronic	Excessively thick subcutaneous fat layer	35 months	Intraoperative findings, patient history	Creating a new subcutaneous pocket at a shallower layer

*DBS, deep brain stimulation; r-IPG, rechargeable implanted pulse generator.*

**FIGURE 1:**
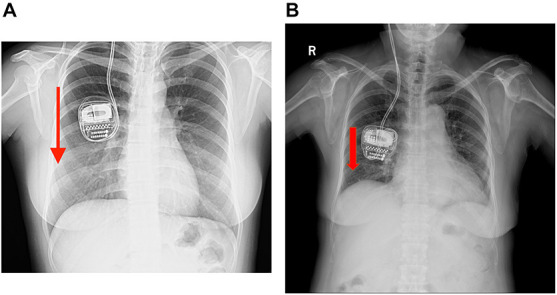
**(A,B)** Plain chest X-rays of cases 1 and 2 showing the upside-down implanted pulse generators (IPGs) but with the extension wires still intact and no evidence of twisting observed. The red arrow means that the r-IPG was upside down.

Case 1 was a 32-year-old female with a long history of severe, medication-refractory Tourette syndrome. Eye blinking and vocal tics were predominant features and cannot be well controlled by optimal medication therapy. During a battery of preoperative neuropsychiatric tests, she reported a history of obsessive–compulsive disorder and anxiety. Bilateral globus pallidus internus (GPi) DBS surgery was recommended to her and implantation of the Activa RC in the right sub-clavicular pocket was performed. Good symptom control was achieved over a 1-year follow-up period. However, 18 months after DBS surgery, she reported experiencing difficulties in charging her r-IPG. When she attempted to use the charger located superficially on the r-IPG, one or two markers lit up on the charger’s screen, implying that perfect coupling between the charger and the r-IPG was not remotely achieved. A plain X-ray (Figure 1A) examination revealed that the r-IPG was upside down inside the chest pocket. During the 18 months postoperative, she had lost more than 15 kg of weight and admitted intentional manipulation of her r-IPG. Manual manipulation by doctors failed to flip the r-IPG. She was then provided with revision surgery. During the operation, an excessively large pocket for the r-IPG was noted, and the r-IPG was found upside down inside the chest pocket. We anchored the r-IPG to the pectoralis fascia with 2–0 silk sutures and reduced the pocket size by stitching its inferior, medial, and lateral aspects. The postoperative period was uneventful. No charging difficulties occurred since then.

Case 2 was a 71-year-old woman who had a history of PD of more than 11 years. At the first visit, her symptoms were a prominent right-sided resting tremor and a gait disorder, wherein she presented with split steps and suffered great postural instability. Bilateral DBS surgery targeting the GPi was performed and the Activa RC was implanted in the right sub-clavicular pocket. No surgery-related complication was encountered. During the following 12 months of follow-up, she had good tremor control and was satisfied with the overall clinical outcome. However, at the 13-month post-operation, she complained of difficulties in charging her r-IPG. The issue of charging difficulty was similar to case 1, wherein one or two markers lit up on the charger’s screen. A plain chest X-ray examination (Figure 1B) revealed an upside-down r-IPG, without evidence of twisting or breakage of the extension wire. Manual manipulation failed to flip the r-IPG. Surgery was recommended to detect any causes of this problem. Intraoperatively, we found that the anchoring wire had fallen and the r-IPG was upside down. We reimplanted the r-IPG and used two anchoring wires to attach it to the pectoralis fascia. She experienced a complication-free recovery.

**FIGURE 2:**
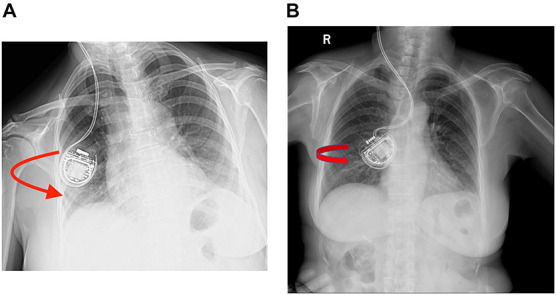
**(A,B)** Plain chest X-rays showing that the rechargeable implanted pulse generators (r-IPGs) were inside out. No fracturing or twisting of the leads was observed. The red arrow means that the r-IPG was inside out.

Case 3 was a 66-year-old woman with a 15-year history of a right-side prominent PD. The preoperative levodopa equivalent daily dose (LEDD) was 600 mg/day, and she complained of peak dose dyskinesia and motor fluctuations. DBS surgery was performed and bilateral electrodes were implanted in the GPi and the G102R model in the sub-clavicular pocket. A test stimulation revealed satisfying outcome, and 3 days later, the dual-channel r-IPG was implanted in the right subcutaneous pocket. Impedance was within the normal range and she was discharged subsequently. However, 2 weeks after surgery, she complained of having difficulties charging her r-IPG. Initially, it was presumed that she did not spot the charger at the right place when charging her IPG, so a detailed explanation of the IPG charging procedure was given to her. However, 1 month later, she visited the clinic again and complained that the charging problem did not improve at all. A plain X-ray examination (Figure 2A) of the patient’s chest, neck, and head revealed that the r-IPG was flipped around. Doctors tried to manipulate the IPG to its correct orientation, but failed because of less mobility. A revision surgery was provided. Intraoperatively, the r-IPG was found to be inside out, but the anchoring wires were in good condition. It is presumed that the r-IPG was implanted in the wrong orientation during the initial surgery. The r-IPG was reinserted and anchored with 2–0 wires. Subsequent follow-ups were free of any complications.

Case 4 was a 69-year-old woman with PD who had similar clinical characteristics and outcome to case 3. Bilateral DBS surgery targeting GPi was performed and the G102R was implanted in the right sub-clavicular pocket. She had an uneventful recovery and was discharged soon after surgery. However, 3 days after surgery, she complained of charging difficulties. A plain chest X-ray examination (Figure 2B) revealed the same result as that of case 2: the r-IPG was found inside out. Manual manipulation failed to flip the IPG. Revision surgery was performed and the r-IPG was reinserted in the correct orientation; no charging difficulties were encountered by the patient since then.

**FIGURE 3:**
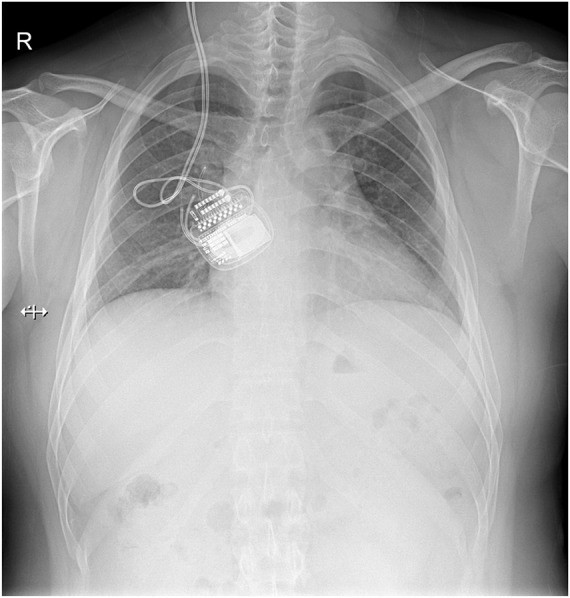
Plain chest X-ray showing the normal view of the implanted pulse generator (IPG) and the extension wire. No fracturing or twisting of the wire was observed and the IPG was in the correct orientation.

**FIGURE 4:**
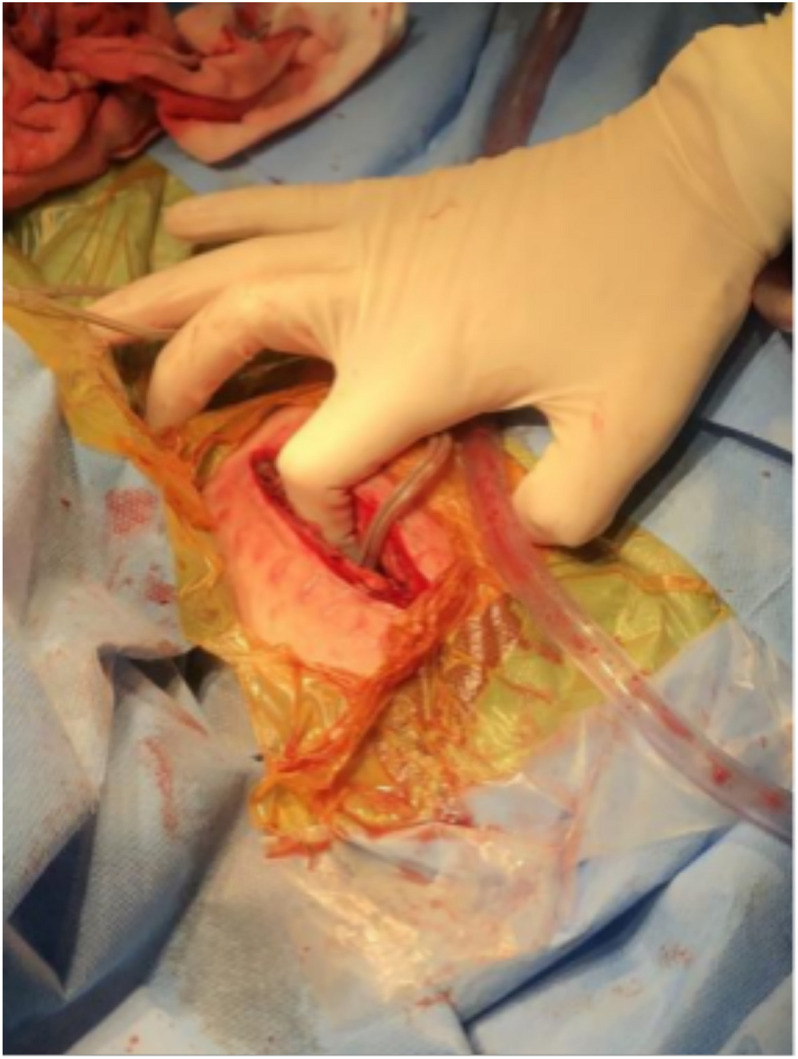
Intraoperative figure showing the thickness of the subcutaneous fat layer which is equal to the length of the index finger to the proximal interphalangeal joint.

Case 5, the last patient, was a 25-year-old man with a long-standing history of Tourette syndrome. He was presented to our hospital in 2016. Before DBS surgery, he was 178 cm tall, weighed 87 kg, and had a body mass index of 27.4 kg/m^2^. Bilateral globus pallidus internus was selected as the DBS target. Subsequently, the Activa RC was implanted in the right sub-clavicular pocket. In 2019, 3 years after the implantation of the DBS system, he began to experience difficulties with charging his r-IPG. He complained of extensive charging time and reduced charging efficiency. When he visited our hospital the second time, he weighed 106 kg and had a body mass index of 33.4 kg/m^2^ (a body mass index >30 kg/m^2^ is classified as obese). The r-IPG was in good position on chest X-ray examination (Figure 3). Revision surgery was then undertaken. During the operation, we found that the thickness of his thoracic subcutaneous fat layer was approximately 2 cm (Figure 4). We removed the r-IPG from the subcutaneous pocket and achieved good connection between the charger and the r-IPG. A shallower pocket, 1 cm underneath the skin surface, was created to accommodate the r-IPG. The charging capability of the r-IPG was restored without complications to date.

## Discussion

The hardware complication was first described by Chelvarajah et al. (2012) when patients struggled to charge their r-IPGs after switching from non-rechargeable IPGs to r-IPGs. This problem arose when the adapter, which is used to connect the preexisting extension cable to the r-IPG, migrated to the surface of the r-IPG and acted as a physical barrier between the r-IPG and the charger, thus obstructing the wireless connection between them. This problem can be resolved either by manual manipulation, wherein the adapter is moved to the deep surface of the IPG, or surgical treatment when manual manipulation fails. Table 1 summarizes two such cases presented in the literature along with the cases presented in this article.

From January 2012 to January 2021, we performed DBS surgeries on 1,226 patients, and charging difficulty occurred in five of them, which means that this complication did occur in 0.4% of patients with r-IPGs. The five cases presented with common characteristic features: reduced charging efficiency, prolonged charging time, and normal impedance under interrogation. Only one or two markers lit up on the charger’s screen, indicating that a connection was not achieved. The complications in four out of five patients were evident under radiographs, presenting with an r-IPG with an incorrect orientation. Manual manipulation failed in the four patients whose r-IPGs were in the wrong orientation under X-ray examination.

To the best of our knowledge, this is the second report of the charging difficulties of r-IPG post-operation, but the first involving various causes such as obesity, weight loss, and wrong orientation of the r-IPG at insertion. Due to excessive weight loss postoperatively, the subcutaneous fat layer became thin and the pocket size enlarged, allowing overt mobility of the r-IPG, which has led to flipping inside the sub-clavicular pocket. Moreover, we presume that neuropsychological factors may also play a crucial role in the induction of this complication. Patient 1 admitted having manipulated her r-IPG multiple times because of feelings of itchiness and failed to move it back to the original position. Previous studies have also revealed that compulsive manipulation of the r-IPG caused rotation of the stimulator and hardware failure, which is called twiddler syndrome (Machado et al., 2005; Pourfar et al., 2015). For case 2, we propose that the r-IPG was improperly anchored during the initial surgery and the anchoring wire loosened after surgery, resulting in a r-IPG that flipped over unintentionally. An interesting observation was the phenomenon of the r-IPG inserted at the wrong orientation during the initial surgery, with the side engraved with the model number facing inward rather than outward. Charging difficulties were experienced when charging the r-IPG was not successful after the initial surgery. For case 5, we suppose that the sub-clavicular adipose layer has increased the physical distance between the r-IPG and the remote charger, thus making it difficult to charge the r-IPG. According to previous publications, obesity could also be a risk factor for twiddler syndrome (Boyle et al., 1998; Burdick et al., 2010; Gelabert-Gonzalez et al., 2010).

All of the patients who experienced charging difficulties were treated with a second surgery wherein the r-IPG was reinserted with stronger fixing measures. To minimize the risk of such IPG-related complications, we propose strategies that use double-anchoring wires to attach the r-IPG to the pectoralis fascia. Attaching the r-IPG to the clavicle and the fat layer should be avoided. A previous study has suggested that single anchoring to attach the IPG may be a predisposing factor for excessive IPG mobility, which could be a risk factor for twiddler syndrome, and proposed that the r-IPG be anchored with double-fixing wires (Sobstyl et al., 2017). Moreover, a detailed explanation should be given to the patients and their caregivers that manual manipulation of the IPG should be strictly prohibited. We anticipate further advancements in r-IPG technology, and subsequently, the day when charging the r-IPG is possible regardless of its orientation.

## Conclusion

The introduction of the new r-IPGs has resulted in a novel hardware complication, wherein charging the r-IPG has been difficult. We discussed five patients who presented with this complication, identified the underlying causes of this phenomenon, and described prevention strategies to reduce instances of this complication.

## Data Availability Statement

The original contributions presented in the study are included in the article/supplementary material, further inquiries can be directed to the corresponding authors.

## Ethics Statement

The studies involving human participants were reviewed and approved by the Ruijin Hospital. Written informed consent was obtained from all participants for their participation in this study.

## Author Contributions

HL, DL, BS, and YP performed the surgery. HL, DS, XX, YL, and CZ wrote the manuscript. All authors contributed to the article and approved the submitted version.

## Conflict of Interest

The authors declare that the research was conducted in the absence of any commercial or financial relationships that could be construed as a potential conflict of interest.

## Publisher’s Note

All claims expressed in this article are solely those of the authors and do not necessarily represent those of their affiliated organizations, or those of the publisher, the editors and the reviewers. Any product that may be evaluated in this article, or claim that may be made by its manufacturer, is not guaranteed or endorsed by the publisher.
